# Efficacy of preharvest application of biocontrol agents against gray mold in grapevine

**DOI:** 10.3389/fpls.2023.1154370

**Published:** 2023-03-13

**Authors:** Valeria Altieri, Vittorio Rossi, Giorgia Fedele

**Affiliations:** Department of Sustainable Crop Production (DI.PRO.VE.S.), Università Cattolica del Sacro Cuore, Piacenza, Italy

**Keywords:** biological control, biological control agents, microorganisms, gray mold, *Botrytis cinerea*, weather conditions

## Abstract

The use of biocontrol agents (BCAs) represents a promising alternative to conventional methods for the management of gray mold in vineyards during the berry ripening stage. The main advantages of BCAs are the short preharvest interval and lack of chemical fungicide residues in wine. In this study, eight commercial BCAs (based on different *Bacillus* or *Trichoderma* species and strains, *Aureobasidium pullulans*, *Metschnikowia fructicola*, and *Pythium oligandrum*) and a reference fungicide (boscalid) were applied to a vineyard during berry ripening over three seasons to evaluate the dynamics over time in terms of their relative efficacies in gray mold control. At 1–13 days after application of BCAs to the berry surfaces in field conditions, the berries were collected and artificially inoculated with conidia of *Botrytis cinerea* under controlled laboratory conditions, and gray mold severity was observed after 7 days of incubation. Significant differences were observed in gray mold severity among years, according to the number of days the BCAs grew on the berry surface before *B. cinerea* inoculation, and the season by day interaction (altogether accounting for >80% of the experimental variance). The variability in BCA efficacy was closely related to the environmental conditions at the time of application and in the following days. Overall, the BCA efficacy increased with the degree days accumulated between BCA application in the vineyard and *B. cinerea* inoculation in the dry (no rain) periods (r = 0.914, P = 0.001). Rainfall and the associated drop in temperature caused a relevant reduction of BCA efficacy. These results demonstrate that BCAs are an effective alternative to conventional chemicals for the preharvest control of gray mold in vineyards. However, environmental conditions can considerably affect the BCA efficacy.

## 1 Introduction

Gray mold, caused by the fungus *Botrytis cinerea* Pers. Fr. (teleomorph *Botryotinia fuckeliana* (de Bary) Whetzel), is one of the main diseases of grapevine (Elad et al., 2016). Although *B. cinerea* develops as a saprophyte, necrotroph, or parasite on multiple grape organs and has multiple infection pathways (Elmer and Michailides, 2007; Elad et al., 2016), the infections occurring during the berry-ripening stage are considered to induce the most severe damage with respect to both the grape yield and quality (Elad et al., 2016). From veraison to harvest [i.e., from growth stage (GS) 81 to GS89 of Lorenz et al. (1995)], the environmental conditions are often favorable for the development of *B. cinerea* (Ciliberti et al., 2015), and the associated biochemical and structural changes make the berries particularly susceptible to direct infection and/or mold development in berries that have been exposed in previous stages and harbor latent infection (Kosuge and Hewitt, 1964; Mundy and Beresford, 2007; Deytieux-Belleau et al., 2009; Tyson et al., 2022). Gray mold on ripening berries can result from the following infection pathways: (i) latent infections established during flowering, becoming visible as rotted berries; (ii) berry infection caused by the conidia produced by the mycelium colonizing the bunch trash (i.e., calyptras, dead stamens, aborted flowers and berries, and tendrils); (iii) direct berry infection caused by wind-dispersed conidia; and (iv) berry-to-berry infection caused by the aerial mycelium produced on adjacent infected berries within the cluster (Elmer and Michailides, 2007; González-Domínguez et al., 2015).

The routine calendar application of chemical fungicides at four specific grape growth stages (González-Domínguez et al., 2019) includes one application at veraison (GS83) and one application before harvest (GS89) for controlling the disease on ripening berries. In recent years, these late-season fungicide treatments have been subjected to increasing limitations to reduce or eliminate chemical residues on grapes and, consequently, in wine (Verger and Boobis, 2013). With increased recognition of their negative effects on the environment and human health (Alavanja et al., 2004; Komárek et al., 2010; Epstein, 2014), as well as the acquired resistance of *B. cinerea* populations to most chemical fungicides (Leroux, 2007; Fernández-Ortuño et al., 2016), there is enhanced interest in seeking environmentally friendly and safe alternatives for gray mold control (Tracy, 2014).

The use of non-pathogenic microorganisms as biocontrol agents (BCAs) is considered a promising alternative for the management of gray mold (Elmer and Reglinski, 2006; Pertot et al., 2017; Fedele et al., 2020b), and the number of commercially available BCAs has increased in recent years (Nicot et al., 2016; EU Pesticides Database, 2022). BCAs have a low impact on human health and the environment and are useful in anti-resistant strategies because they can control *B. cinerea* through various modes of action (MoAs), including competition for nutrients and space, antibiosis, parasitism, and resistance induced in the host plant (Corkley et al., 2022). BCAs also have a short preharvest interval, allowing their application close to harvest. However, BCAs often show lower efficacy and higher variability in control compared with those of synthetic fungicides under field conditions (Tracy, 2014). The reasons for this low and variable gray mold control are related to a complexity of factors that determine the variability in the effectiveness of BCAs between seasons and according to changes in agronomic conditions (Tracy, 2014), and are strictly linked with the routine phenological application of BCAs in the same manner used for synthetic fungicides (Fedele et al., 2020e).

To overcome these limitations and improve their effectiveness, an improved application strategy of BCAs is their use as part of an integrated management approach, in which the combination of different agronomic practices can reduce the potential *B. cinerea* infection risk (González-Domínguez et al., 2020). Moreover, considering that BCAs are living microorganisms, the conditions on the surface of the plant must be favorable for their development and colonization of the target substrate, as well as for the activation of the metabolic pathways that determine their effectiveness (e.g., the production of antifungal metabolites) (Fedele et al., 2020d).

Using a model simulation approach, Fedele et al. (2020a) found that more than 90% of the variability of BCA efficacy was accounted for by the duration of the BCA colonization period, concomitant environmental conditions, and the response of each BCA to the environment (in terms of growth and survival). To validate these simulated results and to better understand the preventative efficacy of various BCAs, in the present study, eight commercial BCAs were applied in a vineyard during the berry-ripening stage and left to grow in the field for 1–13 days. The dynamics in their efficacy for controlling gray mold were then evaluated in an artificial infection experiment in the laboratory for the assessment of gray mold severity. We hypothesized that the efficacy would vary over time after treatment depending on BCA growth (i.e., the longer the BCA grew on the berry surface, the higher the preventative efficacy), and that weather conditions would affect the efficacy dynamics; for this reason, we repeated our experiment over 3 years with contrasting weather conditions. This study can thus offer valuable reference data for identifying the conditions and microorganisms best suited for such an integrated vineyard management approach in utilizing BCAs as an alternative to chemical fungicides.

## 2 Materials and methods

### 2.1 Experimental vineyard and treatments

The study was conducted for 3 years, from 2018 to 2020, in an experimental vineyard located at the campus of Università Cattolica del Sacro Cuore in Piacenza, Italy (Emilia-Romagna region, 45° 2’N, 9° 43’E). The vineyard was planted with the cultivar Merlot, which is known to be highly susceptible to *B. cinerea* (Bisiach et al., 1996). The vines were 6 years old in 2018 and they were trained using the Guyot system; the within- and between-row spacings were 1.2 m and 2 m, respectively. The vineyard was managed following common practices and only fungicides without efficacy against *B. cinerea* were used to control downy and powdery mildews.

Eight commercial BCA products were applied to the vineyard in a complete randomized block design with four replicate plots (six plants per plot); a non-treated control (NT) and a reference fungicide (boscalid, CHEM) were also included. The details of the products are provided in **Table 1** and were applied at the manufacturer-recommended dose using a hand-pump sprayer at the full-ripening stage (GS89) on the following dates: September 7, 2018; August 27, 2019; and September 8, 2020. Hourly data of temperature, relative humidity (RH), wetness duration, and rainfall were recorded by an automated weather station (iMeteos; Pessl Instruments GmbH) located <1 km from the experimental plot. The GS of each vine was assessed weekly in the vineyards according to the scale of Lorenz et al. (1995).

**Table 1:** Plant protection products used in the experiment.

Active ingredient	Commercial product name (acronym)	Active ingredient concentration	Producer	Label dose (g/ha)
*Bacillus amyloliquefaciens* D747	Amylo-X (BAD)	5 × 10^10^ CFU/g	CBC S.r.l.	2000
*Bacillus amyloliquefaciens* FZB24	Taegro (BAF)	1 × 10^10^ CFU/g	Syngenta	370
*Aureobasidium pullulans* DMS 14941-14940	Botector (APD)	2.5 × 10^9^CFU/g (DMS 14941), 2.5 × 10^9^CFU/g (DMS 14940)	Manica S.p.A.	400
*Bacillus subtilis* QST 713	Serenade Max (BSQ)	5.10 × 10^10^ CFU/g	Bayer	3000
*Metschnikowia fructicola* NRRL Y-27328	Noli (MFN)	1–3 × 10^10^ cells/g	Koppert Italia	2000
*Trichoderma atroviride* SC1	Vintec (TAS)	1 × 10^13^ CFU/granul	Belchim S.p.A.	200
*Trichoderma gamsii* ICC 080 *-Trichoderma asperellum* ICC 012	Remedier (TGA)	3 × 10^7^ CFU/g	Gowan Italia	1000
*Pythium oligandrum* M1	Polyversum (POM)	1 × 10^6^ CFU/g	Gowan Italia	250
Boscalid	Cantus (CHEM)		Basf Italia	100

### 2.2 Evaluation of treatment efficacy

To evaluate the efficacy of each treatment, 20 berries (with their pedicels) that did not show any symptoms or signs of rot, visible cracks, or wounds were randomly collected from each replicate at 1, 3, 6, 9, and 13 days after treatment (DAT). The berries were transported to the laboratory in a cooler and placed in metal boxes (20 × 15 cm, with wet filter paper on the bottom) over a metal grid net so that they would not touch each other or the bottom of the box. The berries were inoculated with a conidial suspension of *B. cinerea* isolate 213T, which belongs to the *transposa* sub-species and is characterized by highly aggressive infective properties (Ciliberti et al., 2016). The conidia were obtained from 10-day-old cultures grown on potato dextrose agar at 20°C under a 12-h photoperiod using white and near-ultraviolet (370 nm) light (Black Light UV-A, L18 w/73, OSRAM, Munich, Germany). The conidial suspensions were prepared by flooding the dishes with sterile-distilled water and gently scraping the agar surface with a sterile rod. The suspension was passed through two layers of sterilized cheesecloth (autoclaved at 120°C for 20 min), and the conidia were enumerated with a hemocytometer. The concentration of conidia was finally adjusted to 10^4^/mL by adding double-distilled sterile water. The conidial suspension was uniformly distributed on the berries using a hand sprayer, with 1 mL of the suspension sprayed per box. The boxes were then sealed in plastic bags to maintain a saturated atmosphere and incubated at 25°C and 100% RH with a 12-h photoperiod to favor conidial germination and infection (Ciliberti et al., 2015).

One week after inoculation of *B. cinerea*, the disease incidence was assessed as the percentage of berries showing gray mold symptoms; disease severity was visually assessed as the percentage of the surface of each berry exhibiting gray mold symptoms. The gray mold severity (i.e., assessed for the 20 berries in each replicate box) was finally determined using the standard area diagram of Hill et al. (2010).

### 2.3 Data analysis

Gray mold severity data were subjected to a factorial analysis of variance (ANOVA), in which the factors were year, treatment (the eight BCAs, CHEM, and NT), and the DAT before collection and inoculation with *B. cinerea* (i.e., the BCA colonization period: 1, 3, 6, 9, or 13 days). The gray mold severity (%) data were subjected to arcsine transformation before the ANOVA to conform with the assumption of homogeneous variances among groups. The least-square difference test was carried out to separate means; statistical significance was judged at P < 0.05.

Efficacy (E) was calculated as E = (NT – T)/NT, where NT is the gray mold severity of the non-treated control group and T is the gray mold severity of a specific application of a given test product for a given treatment period at a given time (e.g., the gray mold severity of berries collected in plots treated with CHEM at 3 DAT in 2020).

## 3 Results

The ANOVA showed significant effects of the main factors year, treatment, and DAT (all P < 0.001), which respectively explained 9.1%, 16.5%, and 17.5% of the total variance in gray mold severity. The year × treatment and year × DAT interactions were also significant (P < 0.001), accounting for 10.3% and 28.1% of the total variance in gray mod severity, respectively. However, the treatment × DAT and year × treatment × DAT interactions were not significant (P > 0.05), together accounting for 18.6% of the total variance in gray mold severity. These data showed that differences among years and the time allotted for BCAs to grow on the surface of berries before *B. cinerea* inoculation significantly affected gray mold severity, collectively accounting for 55.5% of the total variance; however, the response of each BCA to these factors had a lower and non-significant effect.

**Figure 1:**
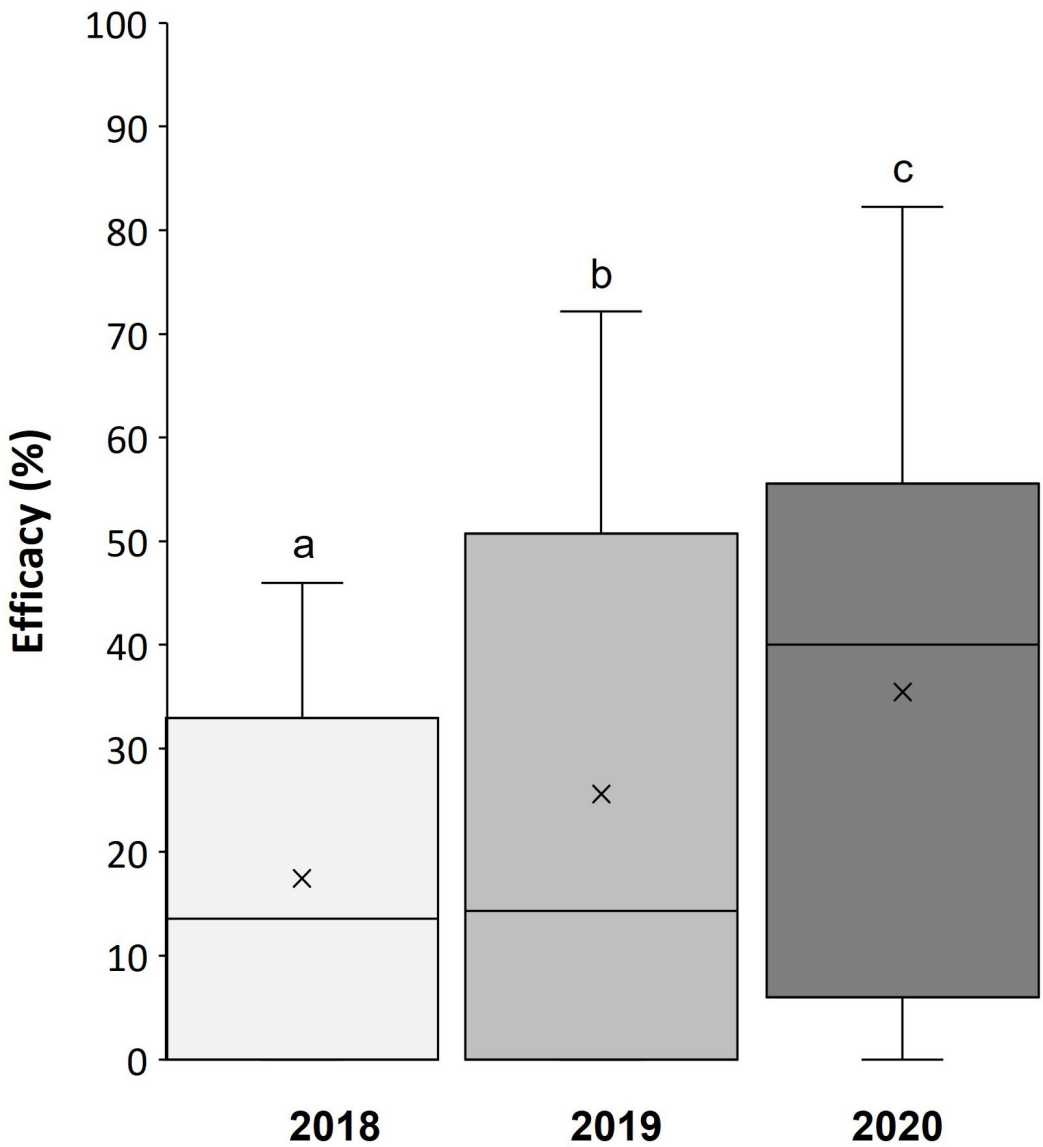
Efficacy (%) of treatments with eight commercial biocontrol agents (BCAs) in reducing gray mold in ripened grapevine berries during three seasons (2018 to 2020). The box extends from the 25^th^ to 75^th^ quartile of the data distribution, the line crossing the box represents the median and × indicates the average; whiskers extend to the maximum and minimum. Efficacy was calculated in relation to an untreated control for berries treated in the vineyard sampled at 1, 3, 6, 9, and 13 days after treatment (DAT), artificially inoculated with a conidial suspension of *Botrytis cinerea* in the laboratory, and then incubated under optimal conditions for the pathogen for one week before disease severity assessment. Each box plot then represents 144 experimental data points (eight BCAs, six DAT, three replicates). Differences between years were significant at P < 0.001 and accounted for 9.1% of the total variance in disease severity. Different lowercase letters indicate significant differences according to the least-square difference test at P < 0.05.

**Figure 2:**
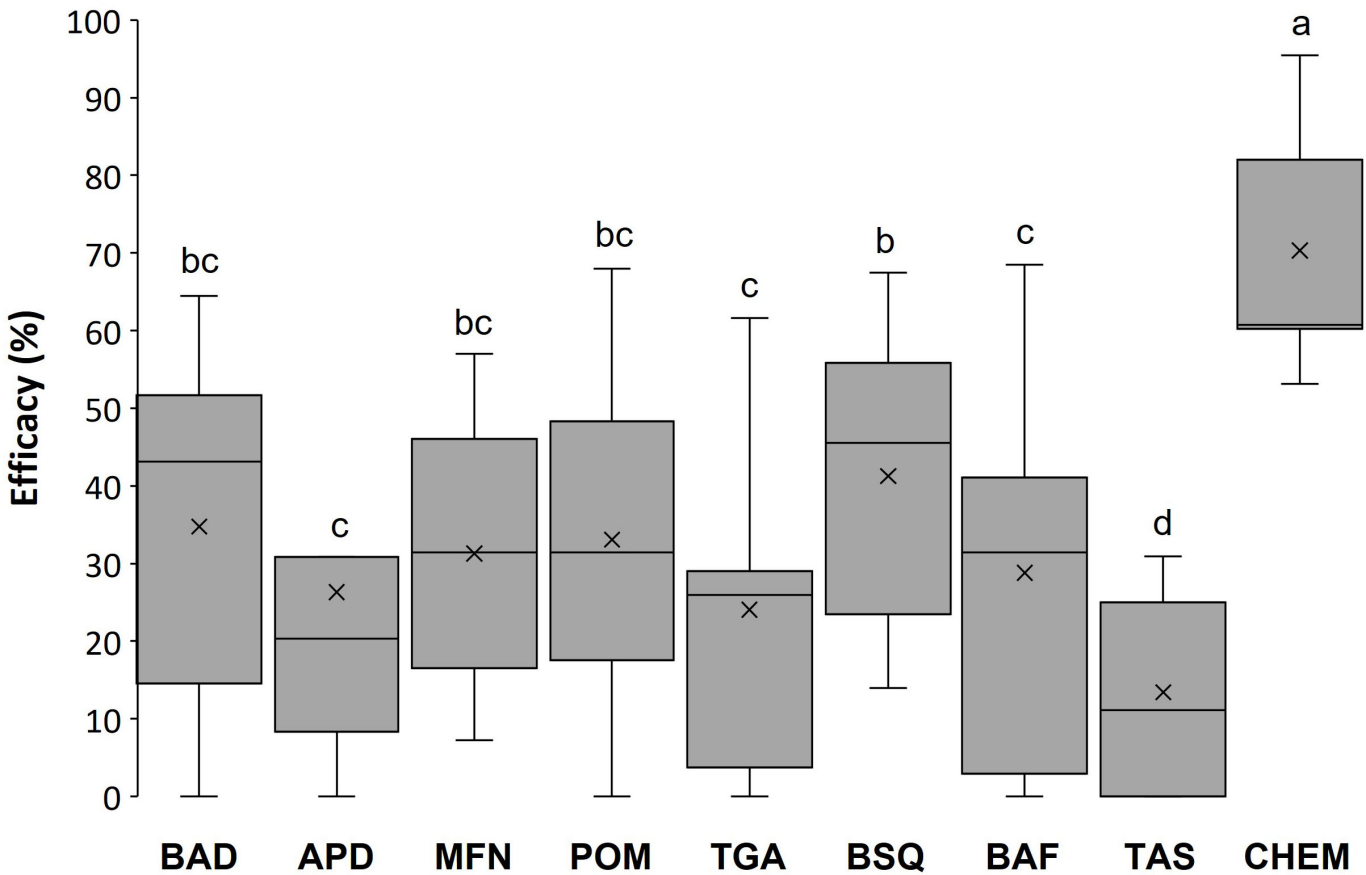
Efficacy (%) of treatments with eight commercial biocontrol agents (BCAs) and a reference fungicide (CHEM) (see **Table 1** for definitions of the acronyms) in reducing gray mold in ripened grapevine berries. The box extends from the 25^th^ to 75^th^ quartile of the data distribution, the line crossing the box represents the median and the × indicates the average; whiskers extend to the maximum and minimum. Efficacy was calculated during three seasons (2018 to 2020) in relation to an untreated control for berries treated in the vineyard sampled 1, 3, 6, 9, and 13 days after treatment (DAT), artificially inoculated with a conidial suspension of *Botrytis cinerea* in the laboratory, and then incubated under optimal conditions for the pathogen for 1 week before disease severity assessment. Each box plot then represents 54 data points (3 years, 6 DAT, three replicates). Differences between treatments were significant at P < 0.001 and accounted for 16.5% of the total variance in disease severity. Different letters indicate significant differences according to the least-square difference test at P < 0.05.

The gray mold severity in the NT group was similar over the 3 years, with 24.7 ± 2.2%, 21.5 ± 2.3%, and 26.6 ± 3.0% of the bunch surface affected in 2018, 2019, and 2020, respectively. The average efficacy of treatments with CHEM was 70.3 ± 10.2%, 62.2 ± 10.4%, and 78.4 ± 10.0% in the three years, respectively; efficacy of BCAs in reducing disease severity was 37.3 ± 4.3%, 25.6 ± 4.4%, and 17.4 ± 3% in 2020, 2019, and 2018, respectively, exhibiting high within-year variability (**Figure 1**). The 3-year average efficacy of different BCAs and of the CHEM control is shown in **Figure 2**, with CHEM exhibiting higher efficacy, than that of all tested BCAs. The efficacy data of each BCA and CHEM in the three years is shown in **Supplementary Material.**

**Figure 3:**
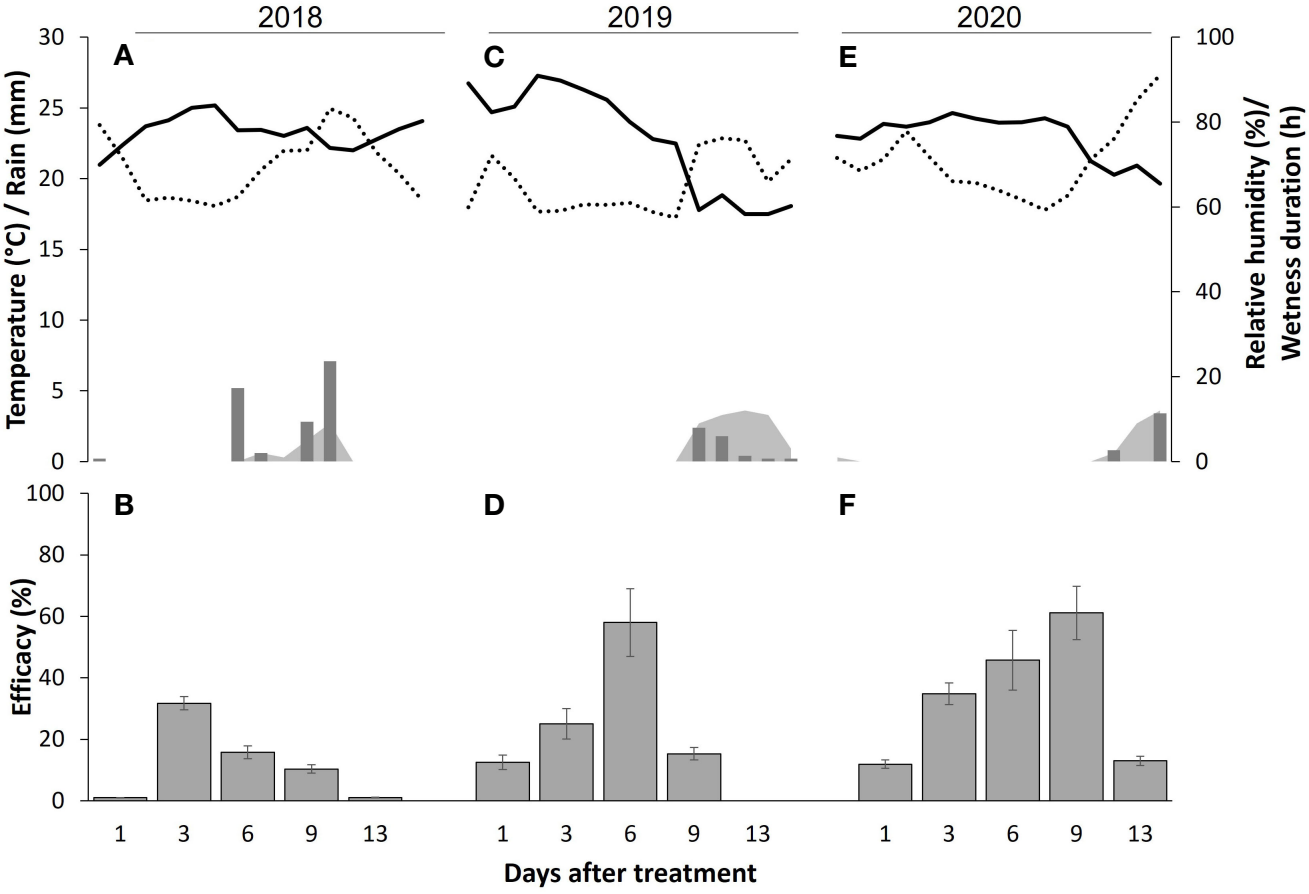
Environmental conditions (upper panel) and efficacy (lower panel). Upper panel: weather conditions recorded in the experimental vineyard in 2018 **(A)**, 2019 **(C)**, and 2020 **(E)**. Daily values of temperature (solid line), relative humidity (dotted line), rain (bars), and wetness duration (gray area) between the day of treatment with biocontrol agents (BCAs) and the following 13 days are shown. Lower panel: Efficacy (%) of treatments in 2018 **(B)**, 2019 **(D)**, and 2020 **(F)** with commercial BCAs in reducing gray mold in ripe grapevine berries at 1 to 13 days after treatment (DAT). Bars are means of eight BCAs and whiskers are standard errors. Efficacy was calculated in relation to an untreated control for berries treated in the vineyard sampled at different DAT, artificially inoculated with a conidial suspension of *Botrytis cinerea* in the laboratory, and then incubated under optimal conditions for the pathogen for 1 week before disease severity assessment. The year × DAT interaction was significant at P < 0.001 and accounted for 28.1% of the total variance in disease severity; the year × treatment × DAT interaction was not significant (P = 0.907).

The year × DAT interaction is shown in **Figure 3**, together with the weather data registered after treatment application. In 2018, the efficacy was almost nil (1 ± 0.1%) when berries were inoculated with *B. cinerea* at 1 DAT, and increased to 31.7 ± 2.2% at 3 DAT (**Figure 3B**); the average temperature in the period between BCA application and berry sampling was 21.7°C [degree days (DDs) accumulated between treatment and DAT, with a basal temperature of 0°C, of 43.4], with 75.5% RH, and increased over the next 2 days (**Figure 3A**). Therefore, the average temperature between application and sampling was 22.8°C (DDs = 91.2), with 68.7% RH. In the following 3 days, the temperature increased again (average 23.6°C; DDs = 164.9, 65.5% RH) and there was a rainfall of 5.2 mm on 6 DAT; as a result, the efficacy decreased to 15.8 ± 2.1%. The following period was also rainy, with a total of 8.8 mm accumulated on 9 DAT (average temperature 23.5°C, DDs = 234.9, 67.4% RH) and 15.9 mm on 13 DAT (average temperature 23.2°C; DDs = 325.4, 69.9% RH); the efficacy decreased to 10.3 ± 1.4% and 1 ± 0.1% on 6 and 9 DAT, respectively.

In 2019, the efficacy of treatments with BCAs progressively increased for berries inoculated with *B. cinerea* at 1, 3, and 6 DAT, with an average of 12.5 ± 2.3%, 25 ± 4.9%, and 58.0 ± 11.0%, respectively (**Figure 3D**). The corresponding average temperatures of these periods were 25.7°C (51.4 DDs), 25.9°C (103.8 DDs), and 26.1°C (182 DDs), which were all higher than those in the corresponding periods of 2018; the average RH in this period was 62.6% (minimum 58.9%, maximum 72.1%), which was lower than that in the previous year (**Figure 3C**). The following period was rainy, with a total of 4.8 mm accumulated, and there was a corresponding drop in temperature (average 20.1°C); the efficacy also decreased to 15.3 ± 1.9% at 9 DAT and was nil at 13 DAT.

In 2020, the dynamics of BCA efficacy were similar to those found for 2019 (**Figure 3F**), with efficacy increasing from 11.9 ± 1.4% at 1 DAT to 61.1 ± 8.7% at 9 DAT in the absence of rain, with a quite constant temperature (average of 23.9°C; range: 22.8–24.6°C) and the DDs increasing from 45.8 to 238.6. Between 9 and 13 DAT, 4.2 mm of rain accumulated and the efficacy decreased to 13.0 ± 1.5% (**Figure 3E**).

**Figure 4:**
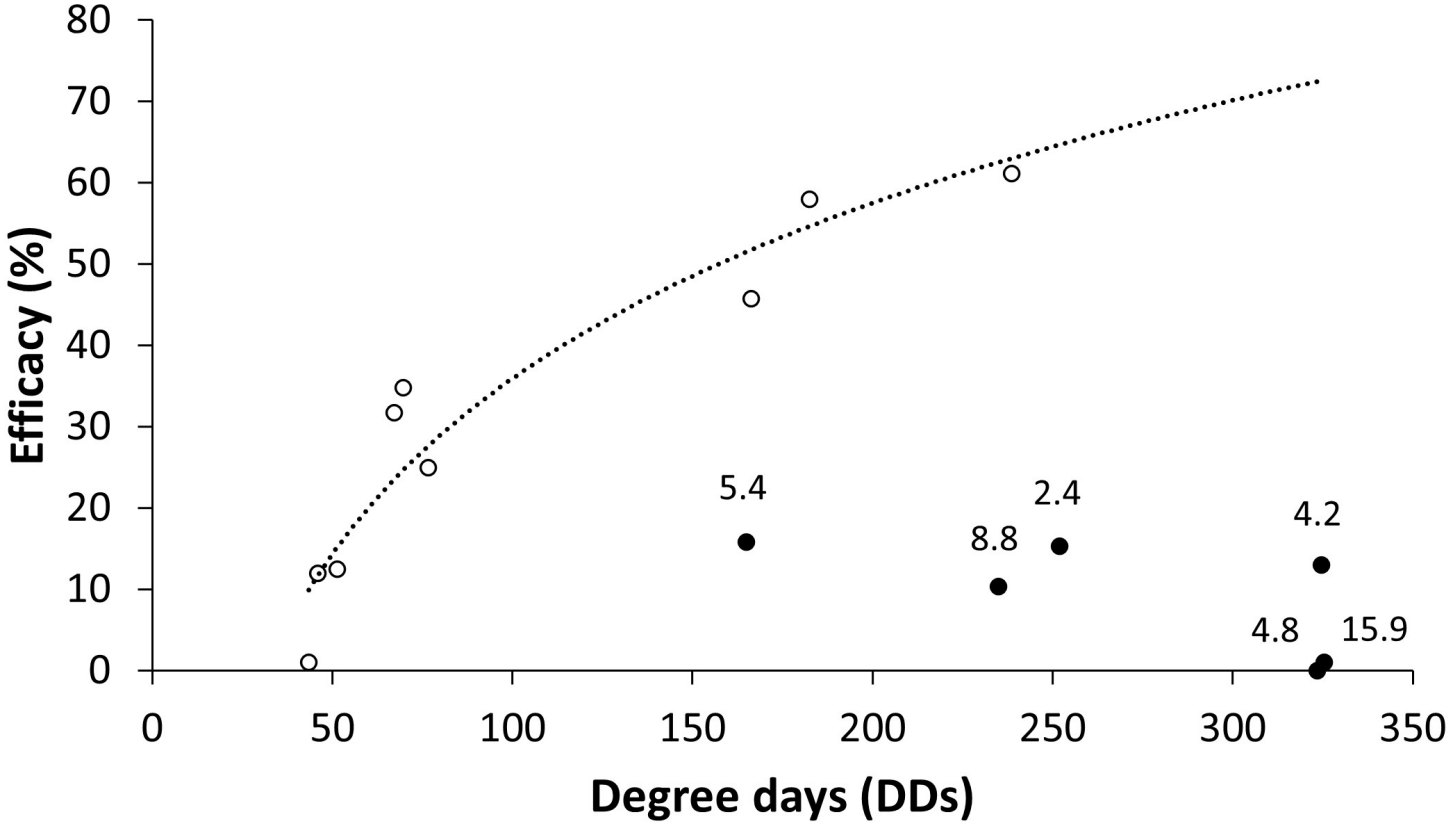
Relationship between the efficacy (%) of treatments with biocontrol agents (BCAs) in reducing gray mold and degree-days (DDs) accumulated from application in the vineyard to the day after treatments when the berries were sampled, artificially inoculated with a conidial suspension of *Botrytis cinerea* in the laboratory, and then incubated under optimal conditions for the pathogen for 1 week before disease severity assessment. Black and white dots represent efficacy in rainy and non-rainy periods, respectively; numbers above black dots show the amount of rain accumulated (in millimeters). The dotted line represents the regression between DDs (x) and efficacy (y) in the non-rainy periods: 31.154ln(x) − 107.56, R^2^ = 0.91. Efficacy was calculated in relation to an untreated control.

Overall, there was a significant relationship between the DDs accumulated after BCA application in the vineyard in the no-rain periods and the BCA efficacy following artificial inoculation with *B. cinerea* (r = 0.914, P = 0.001, n = 9; white dots in **Figure 4**). The BCA efficacy was reduced in rainy periods (black dots in **Figure 4**).

## 4 Discussion

In the present study, we used a reference fungicide (boscalid) and eight commercial BCAs that have shown some efficacy against gray mold under vineyard conditions (Pertot et al., 2017; Alessandri et al., 2018; Rotolo et al., 2018; Calvo-Garrido et al., 2019) to control *B. cinerea* during berry ripening. We focused on this stage because interventions during ripening are commonly considered the most relevant for the protection of bunches in susceptible grapevine varieties (González-Domínguez et al., 2019). This is because berries become more susceptible to *B. cinerea* infection as ripening progresses due to the corresponding changes in pulp composition and in the berry’s skin, including an increase in the concentrations of sugars and assimilable nitrogen, changes in the phenolic compounds in the cell walls of the skin, and the lower water activity on the berry surface (Kosuge and Hewitt, 1964; Deytieux-Belleau et al., 2009; Tyson et al., 2022). The use of BCAs during ripening also constitutes a potential valid alternative to synthetic fungicides because of the shorter preharvest interval, enabling application close to harvest while ensuring the absence of chemical residue in wines (Calvo-Garrido et al., 2019).

During ripening, *B. cinerea* can affect berries by both the conidia and hyphae. Conidia germinate on the berry surface and then produce hyphae that grow epiphytically, which penetrate mainly through microcracks on the berry skin or wounds [i.e., the “conidial infection of ripening fruit pathway” of Elmer and Michailides (2007)]. The mycelium growth outside the surface of affected berries can also infect the adjoining berries through contact [i.e., the “berry-to-berry infection pathway” of Elmer and Michailides (2007) and González-Domínguez et al. (2015)]. Here, we focused only on the conidial infection pathway. Specifically, we applied the BCAs in the vineyard on bunches and then artificially inoculated detached healthy (i.e., with no gray mold symptoms) berries with a conidial suspension of *B. cinerea* under environmentally controlled conditions at different DAT. Preventative application of BCAs is more likely to be effective in preventing conidial germination, growth of germ tubes, and penetration through microcracks compared with controlling the growth of aerial mycelium and consequent berry-to-berry infection (Fedele et al., 2020a).

The preventative efficacy of BCAs on berries is related to their MoA. *Bacillus amyloliquefaciens* subsp. *plantarum* strain D747 (BAD), *Bacillus subtilis* strain QST 713 (BSQ), and *Bacillus amyloliquefaciens* strain FZB24 (BAF) are spore-forming bacteria whose major MoA is antibiosis (Leifert et al., 1995; Borriss, 2015) through the production of lipopeptides, antifungal proteins, lytic enzymes, and volatile compounds (Leifert et al., 1995; Borriss, 2015; Haidar et al., 2016; Calvo-Garrido et al., 2019). *Aureobasidium pullulans* strain DMS 14941-14940 (APD) is a yeast-like fungus that grows as an epiphyte on grape berries and exhibits multiple MoAs. APD colonizes microcracks, forms a biofilm, and produces hydrolytic enzymes (Parafati et al., 2015), thereby competing with *B. cinerea* for the penetration sites; APD also produces volatile organic compounds that prevent the germination of conidia (Di Francesco et al., 2015). The yeast *Metschnikowia fructicola* NRRL Y-27328 (MFN) competes with *B. cinerea* for space (through the formation of a biofilm) and nutrients (Spadaro and Droby, 2016; Millan et al., 2022); for instance, it is able to antagonize the growth of fungi and bacteria in direct competition for iron intake (Sipiczki, 2006). Additional reported MoAs of these yeasts include enzyme secretion, parasitism, and production of volatile organic compounds and metabolites (Spadaro and Droby, 2016; Millan et al., 2022). *Pythium oligandrum* strain M1 (POM) is a mycoparasitic oomycete that attacks the host fungus by lysis or penetration of hyphae (Laing and Deacon, 1991). *Trichoderma atroviride* strain SC1 (TAS), *Trichoderma gamsii* ICC 080, and *Trichoderma asperellum* ICC 012 (TGA) have multiple MoAs, including induction of plant resistance, mycoparasitism, antibiosis, and competition for space and nutrients (Pertot et al., 2013; Chen et al., 2016; Wu et al., 2017).

It is important to consider that although the BCAs were applied in the field and were left to grow on the berries under natural, fluctuating weather conditions for various periods of time, the efficacy results of this study were obtained with artificial inoculation of *B. cinerea* under optimal conditions for infection (with respect to both the inoculum dose and environmental conditions). Artificial inoculation is often used to study relationships among microbial agents and target pathogens (Hannusch and Boland, 1996; Helbig, 2001) as this enables varying only the factors under study while keeping the others constant (Coertze and Holz, 1999). However, a consequence of this experimental setting is that the efficacy levels obtained are not necessarily comparable with those that can be obtained under field conditions, in which the conidial concentration and the environmental conditions are not always as favorable for *B. cinerea*. Indeed, the treatment with the reference fungicide (CHEM) induced an average efficacy of 70.3 ± 7.9% after artificial inoculation, which is lower than the average efficacy reported in a vineyard (Capriotti et al., 2006). However, the main focus of this study was to evaluate the variation of BCA efficacy over time after application rather than its overall level.

We found that more than 80% of the total variance of our experimental data was accounted for by differences among years, time after treatment, and their interactions with the treatment (i.e., the BCA). Accordingly, our results confirmed the theoretical approach of Fedele et al. (2020a) and further supported the conclusion that the wide variability reported in previous field experiments (Pertot et al., 2017; Alessandri et al., 2018; Rotolo et al., 2018; Calvo-Garrido et al., 2019; Carbó et al., 2019), and commonly encountered in practical biocontrol (Magan, 2020), is mainly caused by weather conditions at the time of the intervention and thereafter [see the review of Fedele et al. (2020d)].

In our experiment, the gray mold control efficacy clearly increased with accumulation of DDs after treatment. Our previous study showed that the temperature following BCA application has a key effect on the control efficacy of *B. cinerea* (Fedele et al., 2020b), in which six of the commercial BCAs used in the present study (BAD, APD, POM, BSQ, BAF, and TAS) were applied to grape berries and incubated at different temperature/RH conditions before *B. cinerea* inoculation. Conversely, rain (and the concomitant drop of temperatures) had a detrimental effect on BCA efficacy. Although we did not assess the size of the BCA population on the berry surface at the time of *B. cinerea* inoculation, we can speculate that the time and temperature after BCA application (expressed here as DDs) collectively favored BCA growth and the extent of colonization, whereas the rain washed the BCA off from the berry surface. Rain during the berry-ripening stage can also cause cracks on the berry skin because of the rapid increase in berry turgor due to water absorption (Ramteke et al., 2017). Although we performed *B. cinerea* inoculation on berries that did not show any visible cracks, the presence of microcracks contributing to more severe infection, and consequently lower BCA efficacy, cannot be excluded. However, the wash-off effect of rain is supported by the study of Carbó et al, 2019, who found a decrease in the number of viable BCA cells recovered from berries after a relevant rainfall. Similar observations have been reported by other authors. Metz and Hausladen (2022) and Sylla et al. (2015) observed that abundant rain caused wash off of *Trichoderma* spp. on potato and a settlement reduction of *A. pullulans* on strawberry fruits, respectively. Similarly, Behle et al. (1997) demonstrated the rain wash-off effect on the efficacy of the bacterium *Bacillus thuringiensis* var. Kurstakii against the cabbage looper *Trichoplusia ni*, and Inglis et al. (1995) noted rain wash off of the entomopathogenic fungus *Beauveria bassiana* on alfalfa and wheat crops. Our results thus further support the use of adjuvants to improve BCA survival, population persistence, and better adhesion to the berry surface (Calvo-Garrido et al., 2014; Di Francesco and Mari, 2014; Sare et al., 2021), which can improve the maintenance of BCA efficacy and contribute to reducing between-applications variability. Nevertheless, further studies are needed to better understand the role of rainfall, specifically the relationship between rain amount/intensity and the extent of BCA wash off, and whether the microbial population remaining on the berry surface after rain can recover and ensure some efficacy in gray mold control. Such studies would support the decision as to whether a treatment needs to be repeated following a washing rain.

The results of our work further supplement the results of previous studies in which BCAs have been used to control *B. cinerea* in the early growth stages of grapevine to prevent the latent infection of berries at flowering and the sporulation of bunch trash from flowering onward [i.e., the “conidial infection of floral organs pathways” and the “conidial accumulation pathway” of Elmer and Michailides (2007), respectively]. Flowers, bunch trash, and ripening berries have different characteristics and chemical compositions; thus, different microorganisms used for biocontrol may show different abilities to grow and colonize these different media. Moreover, the activity of BCAs may be different at different vine growth stages: at flowering, BCAs should be able to colonize the flower styles and ovules that *B. cinerea* uses for penetration [i.e., pathway I of Elmer and Michailides (2007)] and prevent infection; from flowering onward, BCAs should be able to permanently colonize the bunch trash and prevent colonization by and sporulation of *B. cinerea* (Fedele et al., 2020e); whereas from veraison to ripening, BCAs should be able to colonize the berry surface and prevent infection, as mentioned above. Based on these interactions, different BCAs have been successfully applied at different vine growth stages to control gray mold (Pertot et al., 2017; Fedele et al., 2020e). Knowledge about the weather conditions affecting BCA efficacy may further improve the appropriate BCA selection at a given time.

The results of our work can then contribute to the development of a decision-making process for BCA applications, which includes the following steps: (i) assess the risk of *B. cinerea* infection using mathematical models (González-Domínguez et al., 2015); (ii) define the BCA candidates that can be used based on the plant substrate (inflorescences, bunch trash, and berries at different ripening stages) at the time of application (Fedele et al., 2020b; Fedele et al., 2020c); and (iii) define the BCA with the highest probability to be effective [i.e., the BCA whose ecological requirements best fit with the weather conditions in the days following application (Loureiro et al., 2012; Fedele et al., 2020b)]. This decision-making process needs further developments and test under vineyard conditions.

## Data availability statement

The raw data supporting the conclusions of this article will be made available by the authors, without undue reservation.

## Author contributions

VR and GF mainly contributed to the conception and the design of the study. GF and VA carried out the experiments and the analysis of results. All authors contributed to the article and approved the submitted version.
