# Supplementary material for: Efficacy of preharvest application of biocontrol agents against gray mold in grapevine
Source: Front Plant Sci. 2023 Mar 13;14:1154370. doi: 10.3389/fpls.2023.1154370 (PMC10040820; doi:10.3389/fpls.2023.1154370)
Supplement: Supplementary file 1 [file DataSheet_1.pdf]

Supplementary material

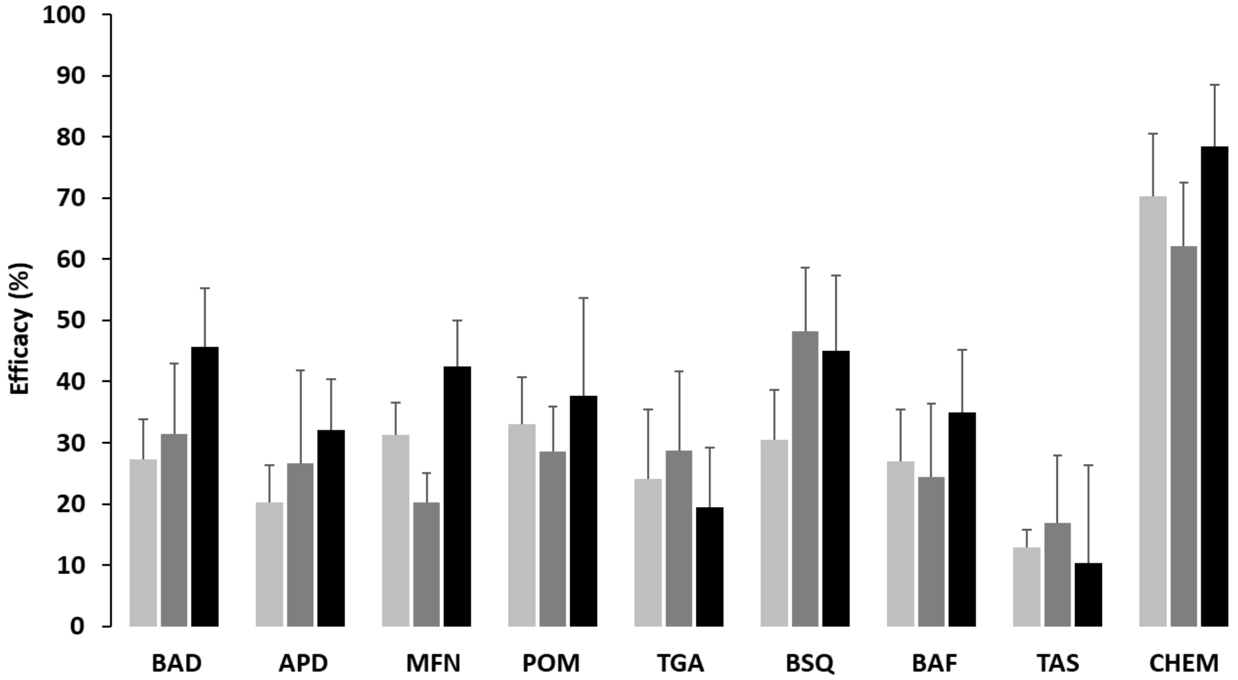

Figure S1. Efficacy (%) of treatments with eight commercial biocontrol agents (BCAs) and a reference fungicide (CHEM) (see Table 1 for definitions of the acronyms) in reducing Botrytis bunch rot in ripen grapevine berries. The bars represent the average efficacy in 2018 (light gray), 2019 (dark gray), and 2020 (black); whiskers are standard errors. Efficacy was calculated in relation to an untreated control for berries treated in the vineyard sampled at different days after treatment, artificially inoculated with a conidial suspension of *Botrytis cinerea* in the laboratory, and then incubated under optimal conditions for the pathogen for 1 week before disease severity assessment.
